# Comparison of Subjective and Objective Sleep Estimations in Patients with Bipolar Disorder and Healthy Control Subjects

**DOI:** 10.1155/2016/4031535

**Published:** 2016-11-06

**Authors:** Philipp S. Ritter, Cathrin Sauer, Steffi Pfeiffer, Michael Bauer, Andrea Pfennig

**Affiliations:** Klinik und Poliklinik für Psychiatrie und Psychotherapie, Universitätsklinikum Carl Gustav Carus an der Technischen Universität Dresden, Fetscherstraße 74, 01307 Dresden, Germany

## Abstract

*Background*. Several studies have described but not formally tested discrepancies between subjective and objective measures of sleep.* Study Objectives*. To test the hypothesis that patients with bipolar disorder display a systematic bias to underestimate sleep duration and overestimate sleep latency.* Methods*. Actimetry was used to assess sleep latency and duration in 49 euthymic participants (bipolar = 21; healthy controls = 28) for 5–7 days. Participants simultaneously recorded estimated sleep duration and sleep latency on a daily basis via an online sleep diary. Group differences in the discrepancy between subjective and objective parameters were calculated using *t*-tests and corrected for multiple comparisons.* Results*. Patients with bipolar disorder significantly underestimated their sleep duration but did not overestimate their sleep latency compared to healthy controls.* Conclusions*. Studies utilizing diaries or questionnaires alone in patients with bipolar disorders may systematically underestimate sleep duration compared to healthy controls. The additional use of objective assessment methods such as actimetry is advisable.

## 1. Introduction

Bipolar disorders are severe mental disorders affecting approximately 1–3% of the population [1]. There is cumulating evidence that bipolar disorder and disturbances of sleep regulation are deeply intertwined on a phenomenological and neurobiological level [2]. Epidemiological research has shown symptoms of insomnia to be a risk factor for the subsequent development of bipolar disorder [3] and various studies have demonstrated patients to suffer from varying degrees of insomnia even when euthymic [4, 5].

Most research regarding sleep in bipolar disorders is conducted using questionnaires or sleep diaries alone [6]. However, there is evidence that patients suffering from bipolar disorder may have deficits in precise time estimation [7] and indeed those studies utilizing objective measures of sleep duration and sleep latency such as actimetry or polysomnography have produced discrepancies between objective and subjective markers with patients often underestimating their sleep duration and overestimating sleep latency [2, 8, 9]. The literature suggests that this discrepancy is most prominent with regard to sleep onset latency and sleep duration. However, while this issue has been exhaustively investigated in patients with primary insomnia [10], there are—to the best of our knowledge—no studies specifically analysing the mismatch of these two parameters in terms of absolute time in bipolar disorder. Assessing the absolute mismatch is important, because the pure correlation of objective and subjective data will reveal a perfect correlation even if there is a systematic mismatch between the two.

We therefore hypothesised that patients suffering from bipolar disorder have a general tendency to underestimate sleep duration and overestimate sleep latency and conducted a study comparing sleep parameters generated by actigraphy with simultaneously completed sleep diaries. The sample utilized was part of a previously published study on early recognition of bipolar disorder [11].

## 2. Methods

We recruited 22 euthymic outpatients from a specialist outpatient clinic for patients with bipolar disorder at the University Hospital Dresden with a diagnosis of bipolar disorder and 28 healthy volunteers via advertisements on campus and other public spaces. One bipolar participant was excluded due to missing diary information.

The recruitment period extends from January 2011 until January 2012.

After providing informed consent, all participants were examined by an experienced psychiatrist using the SCID 1 and 2 [12] for DSM IV as well as the Montgomery-Asberg Depression Rating Scale (MADRS) [13, 14] and Young-Mania Rating Scale (YMRS) [15]. Euthymia was liberally defined as MADRS ≤15 and YMRS ≤10 at screening. The cut-off scores were based on empirical evidence correlating these values to the Clinical Global Impression Scale (CGI-S) in a large cohort of symptomatic bipolar patients. The individual values correspond to a CGI impression of “mildly ill” [16].

Control subjects with first-degree relatives with a major mental disorder except for dementia were excluded. Participants were also excluded if they had a known diagnosed sleep disorder such as narcolepsy, restless leg syndrome, or obstructive-sleep-apnea syndrome. Other medical conditions likely to interfere with sleep (i.e., chronic back pain) and disruptions in circadian rhythms caused by shiftwork, recent (past 3 months) transmeridian flights, or other factors likely to interfere with sleep (i.e., newborn child, significant life events) were considered reasons for exclusion. The presence of symptoms of insomnia was no reason for exclusion.

Participants were required to abstain from the use of illicit drugs for the duration of the study. Sedating medication (i.e., sedating antipsychotics) was permitted if taken regularly for at least one month (Table 2). Patients receiving Benzodiazepines were excluded. The amount of coffee (cups) and alcohol (units) intake was recorded on a daily basis.

The SomnoWatch plus© is microelectromechanical-system (MEMS) based and contains a light sensor and an event marker. All participants wore an actimeter (SomnoWatch plus©, Somnomedics, Germany) for 5 to 7 days and simultaneously completed an online sleep diary (https://www.limesurvey.org/) including questions on estimated sleep duration and estimated sleep latency (Supplement 1 in Supplementary Material available online at http://dx.doi.org/10.1155/2016/4031535). All measurements included one weekend. Participants were required to wear the actimeter at all times except when in contact with water. In the evening the time at which participants lay down to sleep (“lights off”) probands were instructed to press an event marker on the actimeter. The same was required in the morning when participants awoke and wanted to stay awake (“lights on”). The actimeter and algorithm utilized have been validated and show reasonable validity compared to polysomnography [17]. Time in Bed (TIB) is defined as the time between the two event markers. Sleep duration is defined as TIB with sleep latency and wake periods subtracted. The sleep parameters were calculated using DominoLight© software (Somnomedics, Germany). All data was analysed using SPSS© Vs.21.

The self-rating instruments Beck Depression Inventory (BDI) [18] and Altman Self-Rating Scale for Mania (ASRM) [19] were completed as part of the online diary on the first day of assessment.

The differences between estimated sleep parameters and actimetrically determined sleep parameters were calculated on a daily basis for every individual participant (Δ_duration_; Δ_latency_). Group mean and SD were calculated from individual data across the 5–7 days. Group differences were analysed using *t*-test for independent means. The significance level was set at 0.05 and Bonferroni correction for multiple testing was applied.

Normal distribution was assessed using the Kolmogorov-Smirnov test. Differences between groups in coffee and alcohol consumption were calculated using *t*-tests and Pearson correlation to assess association with sleep parameters of interest.

As a possible confounder correlations between age and all related subjective and objective sleep parameters were calculated. In addition, Spearman correlations with ASRM and BDI with subjective and objective sleep parameters were calculated.

The study was approved by the ethics committee of the medical faculty of the University of Dresden (Medizinische Fakultät der TU Dresden).

## 3. Results

Patients with bipolar disorder were approximately four years older on average, though this difference was not significant (*p* = 0.1) (Table 1). The majority of patients had bipolar I disorder (Table 2).

**Figure 1:**
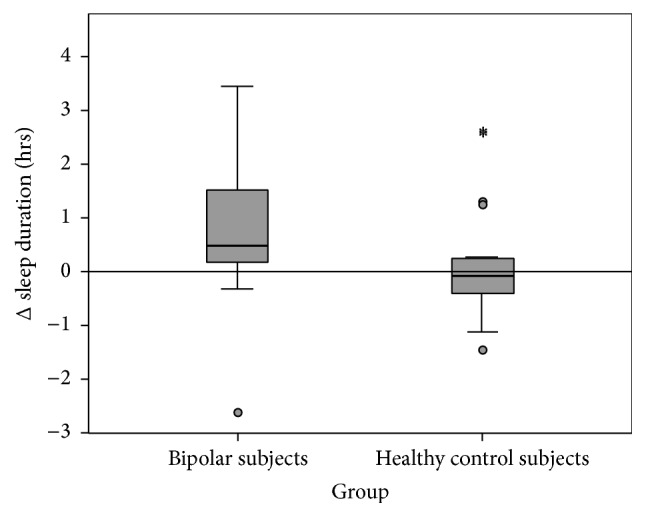
Δ sleep duration. Discrepancy between actimetrically determined values and sleep diary. Positive values indicate subjective underestimation of sleep duration.

**Figure 2:**
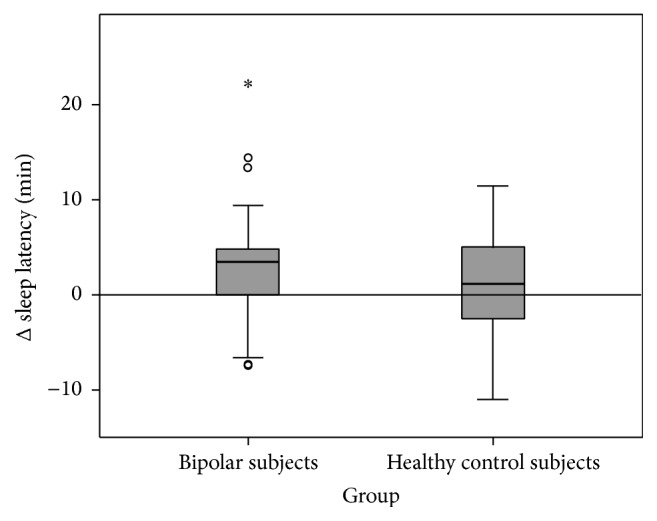
Δ sleep latency. Discrepancy between actimetrically determined values and sleep diary. Positive values indicate subjective overestimation of sleep latency.

Patients with bipolar disorder generally underestimated their sleep duration by 55.05 minutes while healthy control subjects underestimated sleep duration by 4.38 minutes (Figure 1). The between-group difference was significant (corrected *p* = 0.02). Patients with bipolar disorder overestimated their sleep latency by 3.02 min while healthy controls overestimated their sleep latency by 1.12 min (Figure 2). The group differences were not statistically significant (corrected *p* = 0.32).

Approximately a third of participants consumed moderate amounts of alcohol prior to bedtime. The amount of alcohol consumed was slightly higher in the control group (control = 2.93 units/day; bipolar = 2.03 units/day; *p* = 0.40). The amount of coffee consumed was slightly elevated in the bipolar group (control 1.41 cups/day; bipolar 2.01 cups/day; *p* = 0.08). There were no significant correlations between alcohol or coffee consumption and any of the actigraphy or sleep diary parameters of interest.

The BDI score was marginally but significantly elevated in the bipolar group. However, there were no correlations between age, BDI, or ASRM and any of the actigraphy or sleep diary parameters of interest.

## 4. Discussion

The results confirm the observation that patients with bipolar disorder have a systematic tendency to underestimate their sleep duration. They do not appear to misjudge their sleep latency to a significant degree. Previous comparisons of objective and subjective sleep parameters have largely relied on correlations [8, 20]. However while correlating data will give a good measure of covariance it will not detect a systematic tendency to over- or underestimate a certain measure within one group if this over- or underestimation is proportionally constant. To our knowledge, this study is the first to specifically compare absolute values of sleep parameters between patients and controls. A particular strength of this study is the fact that participants were required to estimate their sleep parameters on a daily basis thereby avoiding recall bias.

If the results are confirmed in other samples, certain implications for research on sleep in bipolar disorder will arise. Although diaries are undoubtedly beneficial, convenient, and uncomplicated in characterizing sleep quality overall, estimating sleep duration from diaries or questionnaires alone ought to be avoided especially when making comparisons to healthy controls, because it has to be assumed that patients with bipolar disorder will systematically underestimate their sleep duration. Currently sleep duration is most frequently assessed by questionnaire alone but the results may be biased. This is of particular importance since a substantial portion of the evidence linking bipolar disorder to insomnia is based on questionnaire data. Other methods for assessing sleep duration such as actigraphy have become more available and convenient to use. Due to improvements in memory and battery capacity, many of the currently available devices can assess circadian rhythms and sleep for 7 days or more without recharging and have significantly reduced in size, thus improving suitability for patients. The results should encourage the use of actigraphy or similar technologies for the assessment of sleep duration in bipolar disorder to improve the reliability of results.

The study has several limitations. Firstly, the actimetry settings were very sensitive to optimize detection of sleep latency and wake episodes during sleep. The absolute time of sleep duration may therefore have been slightly underestimated. However there is no reason to believe that this should affect bipolar patients differently from healthy controls. Secondly, the sample size is comparable to other studies utilizing actimetry [21] but larger samples are generally desirable and group differences in Δ_latency_ may not have been detected due to lack of power. Thirdly, participants were allowed to consume coffee and alcohol, which may bias judgement. However no significant differences were found in between groups and overly harsh restrictions on permitted behaviours reduce the external validity of the results. Fourthly, bipolar patients had slightly elevated rates of depressive symptoms. These can certainly impair correct judgement and presumably this also includes duration of sleep. However the absolute level was low and no correlations with the sleep parameters of interest could be found suggesting that there was no systematic bias induced by higher rates of depressive symptoms. Again overly harsh restrictions on psychopathology reduce the external validity, since low grade residual symptoms are common in euthymic bipolar disorder [22]. Fifthly, the use of volunteers always involves the risk of recruiting a sample that is not genuinely representative of the population. There is currently no obvious method to avoid or even quantify this potential bias. Sixthly, the reliability of actimetry improves with duration of measurement, and some [23] but not all authors [24], therefore, recommend a longer duration of measurement. Seventhly, all patients received psychotropic medication. This limitation is applicable to all studies studying sleep or chronobiological aspects of bipolar disorder and it is certainly imaginable that judgement of sleep duration may be altered by psychotropic medication. Since it would be ethically unjustified to discontinue medication and patients with bipolar disorder who regularly attend outpatient clinics commonly receive medication, there is no simple solution to this dilemma. The sample size is too small to study the individual effects of the different medications used.

This study adds to the evidence that patients with bipolar disorder systematically underestimate their sleep duration. Since a multitude of studies have been published on the alterations in the sleep of bipolar patients as assessed by questionnaire these results ought to be evaluated critically. Studies relying on sleep diaries ought to be used with caution in this population and the addition of objective measurements such as actimetry appears to be advisable.

## Supplementary Material

Online-Diary (translated from German).

## Figures and Tables

**Table 1:** Sociodemographic variables.

Group		Bipolar subjects (*n* = 21)	Control subjects (*n* = 28)	Between-group difference *p* value

Age	Mean (SD)	32.95 (10,19)	28.65 (7,3)	0,099^a^
Sex	% female	13 (61,9%)	16 (57,1%)	0,737^b^
Average years in education	Mean (SD)	13.25 (1.53)	13.49 (1.87)	0.64^a^
BDI	Mean (SD)	4.95 (5.7)	0.68 (1.49)	>0.01^a^
ASRM	Mean (SD)	1.5 (1.57)	0.96 (1.55)	0.44^a^

BDI: Beck Depression Inventory; ASRM: Altman Self-Rating Scale for Mania.

^a^ *t*-test; ^b^chi-squared test.

**Table 2:** Clinical variables.

Bipolar subjects		
Age of onset	Mean (SD)	19,38 (7,18)
Number of depressive episodes	Mean (SD)	7,05 (8,67)
Number of manic episodes	Mean (SD)	4,95 (7,17)
Bipolar I/bipolar II	Total	19/2

Medication^*∗*^	Total	%

Lithium	12	57,14
Valproic acid	9	42,86
Lamotrigine	3	14,29
Quetiapine	9	42,86
Other atypical antipsychotics (olanzapine, aripiprazole, clozapine)	4	19,05

^*∗*^Most patients received >1 substance.
