# Supplementary material for: Comparison of Subjective and Objective Sleep Estimations in Patients with Bipolar Disorder and Healthy Control Subjects
Source: Sleep Disord. 2016 Nov 6;2016:4031535. doi: 10.1155/2016/4031535 (PMC5116328; doi:10.1155/2016/4031535)
Supplement: Supplementary file 1 — Online-Diary (translated from German). [file 4031535.f1.pdf]

Supplement 1:

Online-Diary (translated from German)

	**Evening**	Example
**1.**	**Mood?** [1]	6
**2.**	**Were you tired during the day?**	2
**3.**	**How many cups of tea/coffee did you consume?**	5
**4.**	**Did you consume alcohol or sedatives? How much?**	0,5L Bier
**5.**	**What time did you go to bed and try to sleep?**	23:10
**6.**	**How long were you awake for before you fell asleep?**	10 Min
	**Morning**	Beispiel
**7.**	**What time did you wake up?**	6:10
**8.**	**How long did you sleep in total?**	7 Std.
**9.**	**How often were you awake for?**	2x
**10.**	**Are you feeling refreshed/well rested?** [3]	2
**11.**	**Mood?** [1]	3

[1] (sad=1, normal=5, euphoric=10) [2] (not at all=1, very tired=6) [3] (1=fresh and awake, tired/exhausted=6)
